# Medical Review of Reviews, June, 1912

**Published:** 1912-09

**Authors:** 


*Medical Review of Reviews,* June, 1912.

Ophthalmia neonatorum causes 25-40% of all blindness.

There are 6000-7000 persons totally blind in the United States from gonorrhœal ophthalmia.

There are 6200 blind persons in this state, 32% of them caused by this preventable disease.

At the Pennsylvania School for the Blind from 1900-1907, 33 1/3% of the admissions was due to ophthalmia neonatorum.

173 physicians in nine cities in Massachusetts reported an average morbidity rate in infants of 10.8% per thousand births caused by this disease.

One two-hundredth of all births registered in New York State during 1906 represented the toll of blindness due to infection by Neisser's bacillus.

The average cost to the state of a person blind from birth is $10,000.

The Crede method of treatment is effective and there is no excuse for failure to use the 2% silver nitrate solution.

